# Institutional Notes and News

**Published:** 1911-04-29

**Authors:** 


### Institutional Notes and News.

**Serious Fire at Fazakerly City Hospital.**

A serious fire broke out at the Liverpool City Hospital, Fazakerley, on Wednesday last week at 11.30 A.M. and lasted about two hours. No. 1 Ward, a modern single-storeyed building occupied by thirteen children suffering from measles and whooping cough, was completely destroyed. Owing to the prompt action taken by the hospital staff under the supervision of Dr. Rundle, the medical superintendent, all the patients were rescued and also a good proportion of the bedding and furniture. The fire brigade was summoned, and on its arrival some of the firemen entered the burning ward and played on the flames, during which the roof collapsed. The firemen had a miraculous escape, suffering only slight injuries, but it was now found impossible to save the building. The cause of the fire is purely a matter of conjecture, but from the early morning a very severe gale prevailed causing considerable damage to the chimney-pots of this particular ward, and it is thought that the burning soot was blown under the roof, rekindled by the wind, and thus set fire to the rafters. The safety of the other wards was threatened and the wise precaution of emptying the nearest ward was taken, but later the wind turned and carried the flames away. Had this fire occurred at night the result would probably have been disastrous, owing to the firm hold which the fire had gained before it showed any outward sign of its existence. We are informed that fire drills are frequently held at this hospital, and their value was clearly shown by the smartness of the staff last week.

**A Series of Expedients at Doncaster.**

On August 6 last, in this column, we called attention to the adoption of a scheme for building a new infirmary on a new site, by the committee which the Doncaster Royal Infirmary had appointed to consider how the growing demands on its accommodation could best be met. In consequence of this, a New Hospital Fund was started, which has now reached a total of £888. In the meantime the demands for admission have continued to increase far faster than the fund, and this has led to the adoption of a series of desperate expedients to enlarge the accommodation. The Doncaster Royal Infirmary is entered as having forty-three beds, but six months ago the open-air balconies were converted into wards with the help of canvas screens, and nine more beds were thus added to the institution. These were filled immediately, and have remained filled till last week, when the delay caused to patients not merely for admission but in the receiving room itself, has led the committee to take a further step of urgency. This step is the turning out of the nurses from the rooms which they have occupied hitherto at the back of the infirmary's garden—rooms, by the way, originally intended for isolation cases—in order to convert them to patients' uses. For the nurses, accommodation will be found near by in a new house in Cartwright Street, which will be rented by the Board. This plan provides a further eight beds, now bringing the total number up to sixty, and at the same time employs the last resort in the power of the hospital committee. There are now no buildings in which more beds can be placed. Two years ago an extension was made; in the past six years, as a result of the present devices, the accommodation of the hospital has been almost doubled. Collieries have increased enormously in the district of late years, and, as we remarked on a former occasion, the site does not lend itself readily to extension. There is a strong feeling in the district that the workers in the collieries and railway yards are doing much, if not all that they can, to help the institution; but it is also felt that the colliery owners and railway magnates, whose wealth is being produced locally, and for whose workers the hospital largely exists, have not done their fair share for the Doncaster Royal Infirmary. In any case, the above is a summary of the expedients that the infirmary's committee has adopted to meet a situation which want of money has brought to a head by delaying the scheme of reconstruction.

**A Popu'ar Experiment at Cambridge.**

The third quarterly meeting of the Cambridge and District Workers' Hospital Fund was held last week. The number of centres of collection has risen in the past quarter from 82 to 103. Colonel T. W. Harding, the President of the fund, said: "The fund in course of time will have considerable influence in Addenbrooke's Hospital, and the Board will welcome some member as its representative." He again urged that the grant should be made. He also wished the committee of the fund had taken steps in the direction of recommendations to convalescent homes and hospitals, a feature of the work which had made it so popular in every other town. Mr. W. J. Dyball, vice-chairman, urged that the true secret of propaganda was personal spade-work, for there had not been a single firm that had joined without being asked. Their great desire was to get the universities. He had interviewed the heads of different departments to get them to use their influence to get the employees to come in, and they had promised to do their best. We must add that £1,000 hoped for will not be raised this year without great effort, but that if that can be done other southern towns will be greatly encouraged to follow the example of Cambridge. The previous history of the fund will be found summarised in The Hospital of February 11 (page 589).

**Last Sunday's Ceremony at Millbank.**

A large number of patients attended the unveiling of the Memorial to King Edward, which took place on Sunday last at the Queen Alexandra Military Hospital at Millbank. The memorial has taken the form of the redecoration of the sanctuary in the Memorial Chapel, in the south side of which has been placed a credence shelf in alabaster, while to the south chancel-wall an alabaster tablet has been added. The tablet, which was unveiled by Major-General Codrington, commanding the London District, is inscribed: "In grateful remembrance of our Sovereign Lord King Edward the Peacemaker, who reigned from January 22, 1901, to May 6, 1910, and who, with Queen Alexandra, honoured the hospital with visits and gifts and attended the dedication of this chapel on June 24, 1909." The service which accompanied the unveiling was conducted by Bishop Taylor Smith, Chaplain-General to the Forces. The hospital contains 223 beds.

